# Co-Regulation Analysis of Domain-Specific Phosphosites Reveals Insights into the Phosphoregulation-Directed Functional Diversity of Nucleolin

**DOI:** 10.3390/ijms27209132

**Published:** 2026-10-15

**Authors:** Megha Shaji, Jaytha Thomas, Pathiyil Sajini Sekhar, Suhail Subair, Althaf Mahin, Leona Dcunha, Alimath Sambreena, Rajesh Raju, Sowmya Soman

**Affiliations:** Centre for Integrative Omics Data Science (CIODS), Yenepoya (Deemed to Be University), Mangalore 575018, Karnataka, India; meghavellakkal@gmail.com (M.S.); jaythathomas528@gmail.com (J.T.); sajinisekhar333@gmail.com (P.S.S.); suhailsubair.ciods@yenepoya.edu.in (S.S.); 24665@yenepoya.edu.in (A.M.); leonadcunha@gmail.com (L.D.); sambreenaibrahim75@gmail.com (A.S.)

**Keywords:** NCL, phosphoproteomics, phosphosites, chromatin organization, RNA metabolism

## Abstract

Nucleolin (NCL) is a highly phosphorylated, multilocalized, pleiotropic nucleolar protein involved in chromatin organization, DNA damage response, the cell cycle, RNA metabolism, and ribosome biogenesis. A large-scale integration of publicly available human cellular phosphoproteome datasets led to the identification of 18 frequently detected/reported phosphosites across 1266 profiling and 507 differential datasets. Amidst various strategies currently being considered to target NCL in cancers, multi-domain distribution of these phosphosites differentially regulated across distinct cancers highlighted its plausible involvement in tumor-associated regulatory mechanisms. This demands the exploration of the potential kinases and interactors associated with these phosphorylation events. We performed a co-occurrence analysis to explore potential coordinated phosphorylation events among them and across other phosphoproteins. An integrative analysis of phosphosite-specific co-regulation, upstream kinases, and interaction networks was employed to explore potential phosphosite-specific signaling associations. Intriguingly, our analysis inferred the association of the predominant phosphosites within the acidic tract with chromatin organization and histone-related functions, whereas TPxKK motif phosphosites were linked to NCL localization, chromosome segregation, and nucleolar reorganization. Phosphosites in the RRM domain showed a potential association with RNA-binding proteins and metabolic pathways. Collectively, these findings suggest a domain-specific phosphosite-centric regulatory network of NCL, providing a valuable reference framework for future experimental studies and phosphosite-directed therapeutic strategies in cancers.

## 1. Introduction

Histone chaperones are critical for maintaining the dynamic structure of chromatin. They facilitate histone storage, nuclear transport and deposition of histones onto DNA during replication-dependent chromatin organization [1]. Nucleolin (NCL) is a prominent nucleolar histone chaperone that accounts for nearly 10% of the total nucleolar protein content [2,3]. Within the nucleolus, it participates in ribosome production, chromatin remodeling, regulation of transcription and nucleolus organization [3,4].

NCL was first discovered in Novikoff hepatoma cells and Chinese hamster ovary (CHO) cells [5]. It contains 710 amino acids and has a predicted molecular weight of approximately 77 kDa [6]. Structurally, NCL consists of three distinct structural and functional domains: an acidic N-terminal domain, a central domain with four RNA recognition motifs (RRMs) and a C-terminal glycine–arginine-rich domain (RGG or GAR domain) [7]. The N-terminal domain contains four acidic tracts and eight TPxKK repeat motifs. Acidic tracts facilitate histone binding, while TPxKK repeat motifs serve as recognition sites for proline-directed kinases implicated in the localization of NCL. In addition, a bipartite nuclear localization sequence (NLS) located at the end of the N-terminal disordered region (amino acid 279–298) facilitates its transport to the nucleus [8]. The central RRM domain mediates RNA binding and is involved in rRNA processing and telomerase trafficking, whereas the GAR domain is associated with ribosome assembly, nuclear import of ribosomal proteins and protein–protein interactions [5].

Even though NCL is predominantly localized within the nucleolus, it is also distributed across various subnuclear and cellular compartments [8]. In the nucleoplasm, NCL is involved in DNA replication and DNA damage response, including DNA repair, cell-cycle checkpoints and cellular stress responses [3]. NCL continuously shuttles between the nucleus and cytoplasm, where it performs diverse functions, including centrosome duplication and post-transcriptional and translational control of multiple mRNAs such as tumor protein p53 (TP53) [9]. In addition to its intracellular roles, NCL acts as a receptor for many viruses, bacteria and toxins and functions as a cell-surface binding protein for several ligands implicated in angiogenesis and carcinogenesis [8,10].

The regulation of nucleolin is mainly through intracellular localization and post-translational modifications such as phosphorylation, methylation, glycosylation and ADP-ribosylation. Among these, phosphorylation is the major regulatory mechanism [11]. NCL may be phosphorylated by various protein kinases. In actively dividing cells, casein kinase 2 alpha 1 (CK2) may phosphorylate serine residues located within the N-terminal consensus “SEDF” motif during interphase, whereas cyclin-dependent kinase 1 (CDK1) may phosphorylate the threonine residues located in the N-terminal “TPxKK” motif during mitosis [9]. Changes in NCL phosphorylation have been associated with multiple cellular events, including growth and proliferation, DNA replication, regulation of the cell cycle, apoptosis and DNA damage response [4].

The multifunctional role of NCL is closely associated with the development and progression of diseases, including cancer, viral infections, and neurodegenerative disorders [12]. Additionally, we also proposed NCL as a potential primary immunodeficiency disease gene [13]. In cancer, it is frequently overexpressed and often exhibits aberrant intracellular localization [14]. Elevated expression of NCL has been observed across several cancers, including melanoma, colorectal cancer, leukemia, lung cancer, breast cancer, and hepatocellular carcinoma [15]. Moreover, the accumulation of NCL on the cell surface is frequently found in tumors, where it functions as a receptor for multiple ligands involved in tumorigenesis and angiogenesis [10]. NCL has also been implicated in several oncogenic signaling cascades, including PI3K/AKT and RAS-MAPK/ERK. In hepatocellular carcinoma cells, NCL acts as a receptor for HDGF, mediating HDGF-induced PI3K/AKT activation and promoting tumor progression [16]. Recent studies have shown that NCL functions downstream of oncogenic RAS, where ERK1/2-mediated phosphorylation regulates its RNA-binding activity, thereby enhancing ribosome biogenesis and promoting pancreatic cancer [17]. Additionally, several therapeutic approaches have focused on targeting cell surface NCL to inhibit its tumor-associated functions. These approaches include targeting NCL using the AS1411 aptamer, F3 peptides, HB-19, and N6L, which have been explored for their potential to suppress cancer cell proliferation and angiogenic activity and promote apoptosis [6].

Despite the extensive studies on NCL structure, function, and phosphorylation, a comprehensive understanding of signaling networks associated with individual phosphosites remains limited. The current study presents a large-scale phospho-centric analysis of NCL by integrating phosphoproteomic datasets to identify predominant phosphosites of NCL and examine their associated signaling networks. By combining phosphosite-specific co-regulation analysis, upstream kinase prediction, and interaction network mapping, we systematically characterize the signaling networks associated with individual NCL phosphosites. This integrative analysis provides insight into the phosphorylation-dependent regulation of NCL and highlights potential associations between specific phosphorylation events and biological processes, including cell-cycle progression, DNA damage response, RNA metabolism, nucleolar dynamics, and oncogenic signaling.

## 2. Results and Discussion

### 2.1. Global Phosphoproteomic Assembly and Analysis of Phospho-NCL Datasets

To uncover the phosphorylation landscape of NCL, we carried out an initial screening of over 3825 publicly available human phosphoproteomic datasets. This led to the extraction of 1266 profiling datasets containing 60 class-1 NCL phosphosites and 507 differential datasets with 48 class-1 NCL phosphosites, respectively. The entire list of profiling and differential datasets is provided in Supplementary Table S1A,B.

### 2.2. Identification of Predominant NCL Phosphosites

**Figure 1:**
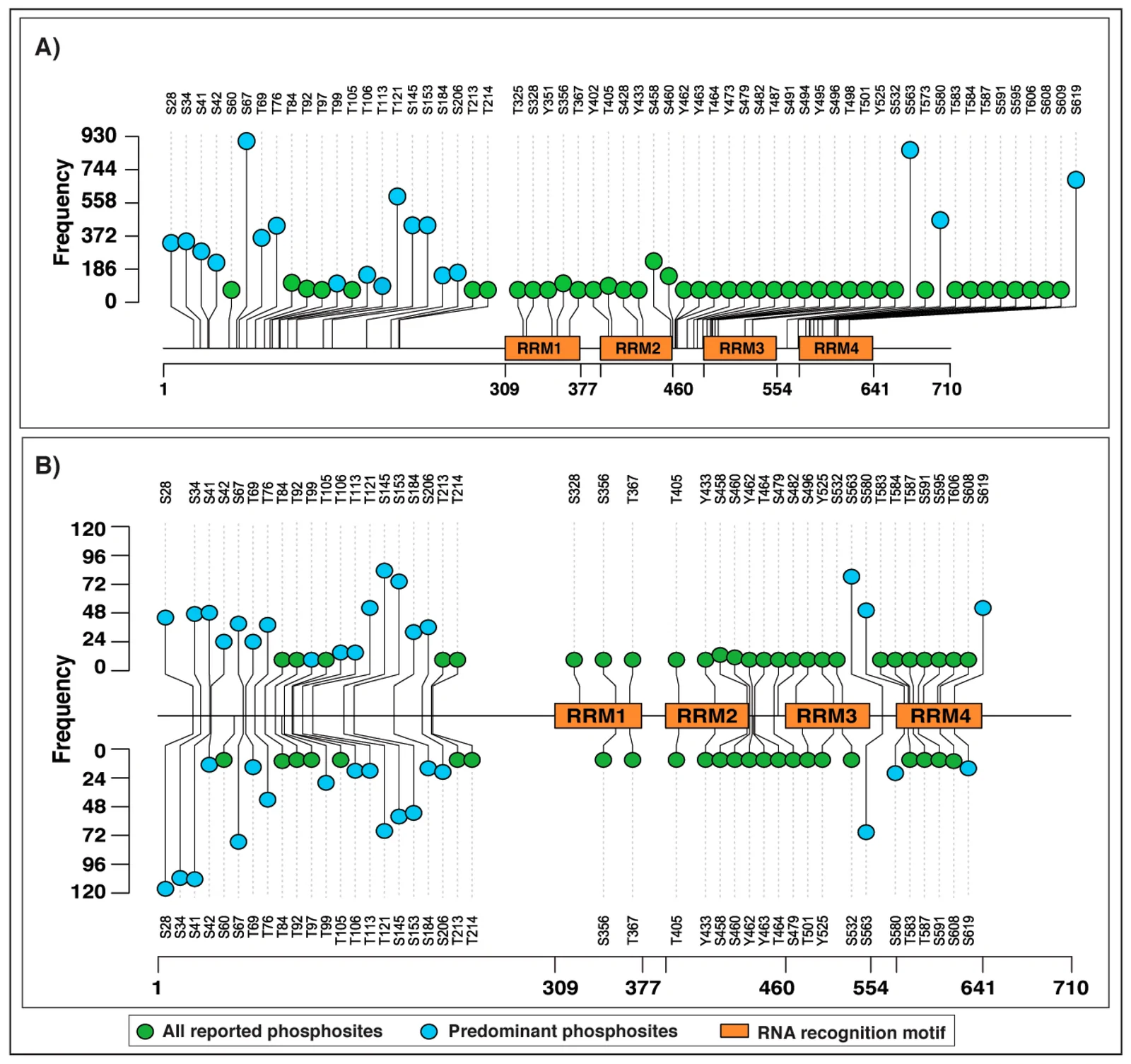
Lollipop plot illustrating NCL phosphosites identified across human phosphoproteomic datasets. (**A**) Representation of phosphosites from profiling datasets. (**B**) Representation of phosphosites from differential datasets.

To determine the predominant phosphosites of NCL, class-1 phosphosites were ranked based on their frequency of occurrence across quantitative differential datasets. Among the 48 differentially regulated phosphosites, 18 phosphosites were consistently detected with significant frequencies. Specifically, S28, S41, S34, S563, S145, S153, T121, S67, T76, S580, S619, S206, S184, T69, S42, T99, T106, and T113 were identified as the most frequently regulated phosphosites occurring in 166, 162, 160, 153, 145, 133, 126, 122, 86, 76, 74, 61, 54, 45, 43, 40, 39, and 39 datasets, respectively. On the basis of their recurrent detection across multiple datasets, these sites were denoted as the predominant NCL phosphosites. The NCL phosphosites detected in profiling and differential datasets are illustrated in Figure 1.

### 2.3. Analysis of Co-Occurring Phosphosites in NCL

**Figure 2:**
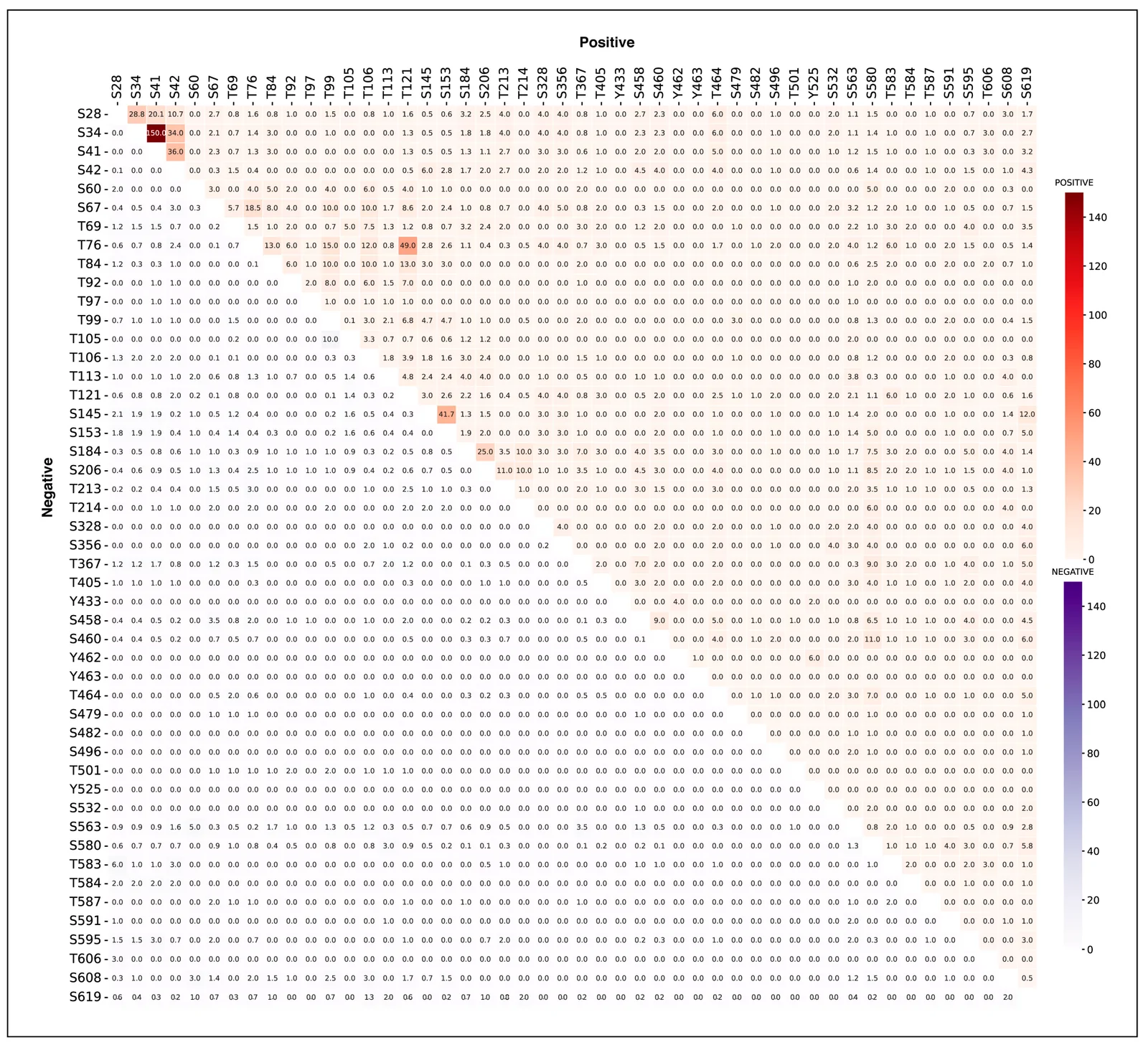
Representation of the co-occurrence patterns among NCL phosphosites. Each cell indicates the degree of co-occurrence between phosphosite pairs within NCL. Red color represents positive co-occurrence, whereas violet color represents negative co-occurrence. The relative co-occurrence ratios across datasets are provided as numerical values.

Phosphosites that co-occur tend to be functionally similar and are evolutionarily conserved [18,19]. Earlier studies have reported that post-translational modifications (PTMs) occurring at different sites within the same protein can cooperate or interact to regulate specific biological functions, a phenomenon referred to as PTM cross-talk [18]. To investigate this context in NCL, we examined co-occurrence patterns of differentially regulated phosphosites (Figure 2). The phosphosite pairs that were concordantly upregulated or downregulated in both were classified as positive co-occurrence, while those exhibiting opposite regulation patterns were considered negative co-occurrence. Interestingly, several predominant NCL phosphosite pairs displayed strong positive co-occurrence across phosphoproteomic datasets. The highest co-occurrence ratio was observed for S41-S34 (150). Similar patterns were observed for other predominant phosphosite pairs, including T121-T76 (49), S153-S145 (41.7), S42-S41 (36), S42-S34 (34), S34-S28 (28.8) and S206-S184 (25). These coordinated phosphorylation patterns suggest a potential functional association between co-occurring predominant phosphosites of NCL. The co-occurrence data of predominant NCL phosphosites are provided in Supplementary Table S2.

### 2.4. Regional Distribution and Functional Characterization of Predominant NCL Phosphosites

**Figure 3:**
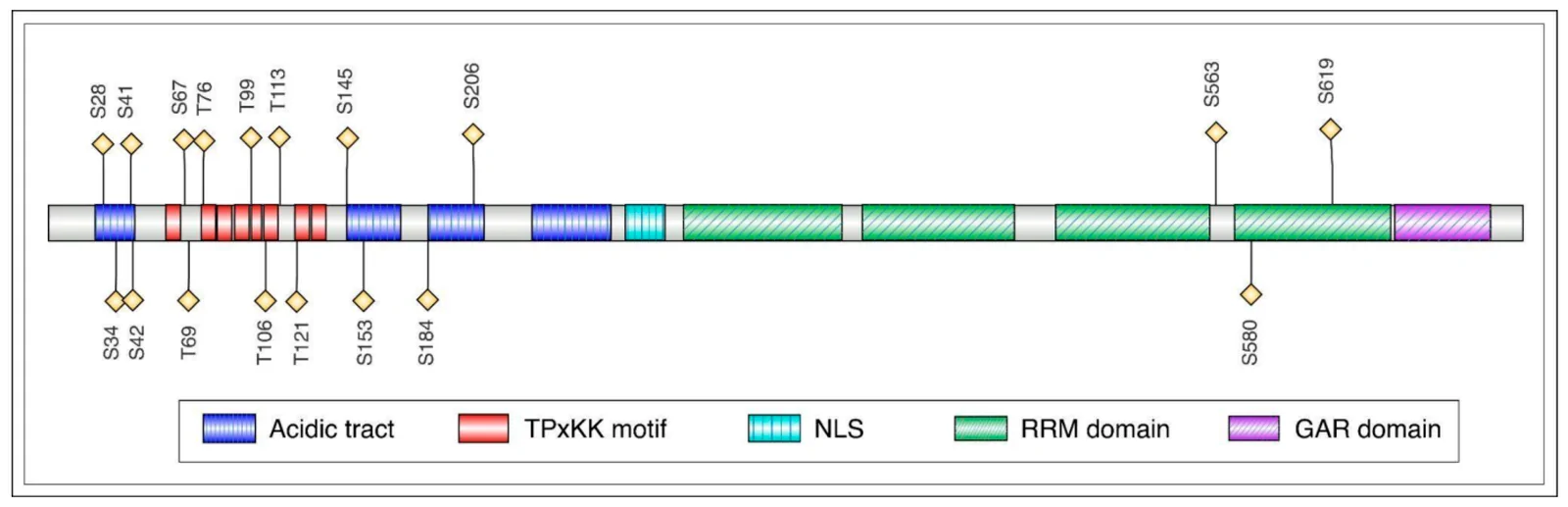
Mapping of predominant NCL phosphosites onto its structural and functional domains.

NCL contains distinct structural and functional domains, including an N-terminal intrinsically disordered region (IDR), a central region composed of four RRMs and a C-terminal IDR enriched in RGG domains. The N-terminal IDR is characterized by four acidic tracts and eight repeated TPxKK motifs [8]. To better understand the regulatory context of predominant phosphosites, the 18 predominant phosphosites were mapped and cataloged according to these structural and functional regions of NCL and categorized into three groups: predominant phosphosites in the acidic tract, predominant phosphosites within and between the TPxKK motif, and predominant phosphosites within and between the RRM domain. The distribution of these phosphosites across distinct structural and functional regions of NCL is provided in Figure 3. Considering that phosphorylation is a key modification of NCL that regulates its intracellular localization and biological functions [9], we further investigated the potential functional significance of these predominant phosphosites through a co-regulation analysis.

#### 2.4.1. Predominant Phosphosites in the Acidic Tract

The N-terminal IDR of NCL contains four acidic tracts, and the longest one consists of 38 uninterrupted aspartic or glutamic acid residues in a row [8]. These acidic tracts can interact with histones and contribute to nucleosome assembly [2]. Among the 18 predominant phosphosites identified in NCL, eight sites (S28, S34, S41, S42, S145, S153, S184, and S206) were clustered within this region, underscoring its regulatory importance. Notably, predominant NCL sites S28, S34, S145, S153, S206, and S184 have been implicated in cell-cycle regulation and cell growth [9].

#### 2.4.2. Predominant Phosphosites Within and Between the TPxKK Motif

The N-terminal IDR contains eight repeats of the [ST]PxK[KA] motif, mostly in the form of TPxKK, which are phosphorylated by proline-directed kinases and are important for NCL localization [8]. These motif regions are separated by linker (inter-motif) regions. Seven predominant phosphosites were identified in this region, including four phosphosites (T121, T76, T99, and T106) located within the motif regions and three phosphosites (S67, T69, and T113) located between the inter-motif regions, highlighting their regulatory importance. Notably, phosphorylation of T76 has previously been associated with cell-cycle regulation and cell growth [20].

#### 2.4.3. Predominant Phosphosites Within and Between the RRM Domain

The central domain of NCL contains four RRM domains (RRM 1, RRM 2, RRM 3 and RRM 4) that facilitate rRNA modification and processing by binding to the stem–loop structure of RNA [5,7]. In addition to these functions, the RRM domain is also involved in G-quadruplex stabilization [21]. Among the 18 predominant phosphosites identified in this study, two phosphosites, S580 and S619, were located within the RRM domain (RRM 4), whereas S563 was located between RRM 3 and RRM 4. The presence of recurrent phosphosites within and between these regions suggests potential regulatory significance.

In order to provide further insights into the potential regulatory and biological significance associated with the identified predominant phosphosites, we performed a co-regulation analysis, upstream kinase predictions, and binary interaction network mapping.

### 2.5. Analysis of Co-Regulated Protein Phosphosites with Predominant NCL Phosphosites

A co-regulation approach was adopted to list the PsOPs that consistently co-regulate with predominant NCL phosphosites. Using the stringent selection criteria detailed in the Materials and Methods, we identified high-confidence co-regulated proteins (Supplementary Table S3). The numbers of positively and negatively co-regulated high-confidence PsOPs corresponding to the respective predominant phosphosites located in different regions are summarized in Table 1. The top 10 high-confidence PsOPs exhibiting positive and negative co-regulation are represented in Supplementary Figure S1.

Co-regulation analysis revealed that PsOPs associated with the predominant phosphosites of NCL were consistent with the known functional roles of the corresponding NCL regions. Specifically, several predominant sites within the acidic tracts exhibited co-regulation with PsOPs involved in chaperone activity and chromatin organization, reflecting the established histone-binding function of this region. Specifically, S41 was positively co-regulated with CHAF1B (T432), a histone chaperone involved in chromatin assembly, while S145 was positively co-regulated with PTGES3 (S113), an HSP90 co-chaperone associated with protein stability, and NASP (S451), a histone chaperone involved in histone homeostasis [22,23,24]. Likewise, S153 exhibited positive co-regulation with MCM2 (T39), a replication-associated protein with histone-binding properties [25].

In addition, S206, S184 and S42 exhibited extensive co-regulation with chaperones and chromatin-organizing PsOPs. For S206, positive co-regulation was observed with CHAF1A (S255), KMT2D (S1180), KMT2B (S632), SETD2 (S323, S1845, S1844, S1417, S321), RSF1 (S604, S629), DOT1L (S826), and SMARCA4 (S1642, S1644, S1640, S1631, S1627), whereas negative co-regulation was observed with CHAF1A (S458), EP400 (S315), JADE1 (S89) and BAZ1A (S1417). Likewise, S184 was positively co-regulated with NASP (S244, S428), ARID4B (S296), SETD2 (S323, S321, S1845, S131), KMT2B (S632) DEK (S230, S231, S232), CHAF1A (S255), SUPT6H (S125) and negatively co-regulated with CHAF1A (S458). S42 displayed positive co-regulation with DOT1L (S834), MBD2 (S407), HMGA2 (S105, S102, S101, S100), BPTF (S216), SSRP1 (S437), HJURP (S140), ATRX (S92, S819) while negatively co-regulated with CHAF1B (S409), RSF1 (S392, T1278), CHD2 (S1373) ARID4B (S666), PSIP1 (S118). Collectively, these findings suggest that phosphorylation of predominant phosphosites within this region is associated with chromatin organization and histone interactions.

Interestingly, compared to acidic tract phosphosites, multiple nucleoporins were found to be enriched (GO:0005643-nuclear pore) and co-regulated with the predominant NCL phosphosites located within the TPxKK motif region (Supplementary Table S6). Nucleoporins are the core structural components of the nuclear pore complex (NPC) that govern the bidirectional transport of proteins and RNAs between the nucleus and cytoplasm [26]. Among these sites, T121 exhibited positive co-regulation with nucleoporins such as NUP98 (S839, T1000), AHCTF1 (T1257), RANBP2 (S2900, S2270, S128, S1613), and NUP133 (T28, S27), while T76 was positively co-regulated with NUP98 (S839), NUP153 (S529), NUP133 (T28, S7), AHCTF1 (S2226, T1257, S2222), TPR (T1677), and RANBP2 (S1613, S1128, S2270, S948, S1869). Likewise, T99 exhibited positive co-regulation with NUP214 (T437, S433), NUP153 (T388, S529), AHCTF1 (S1898, S1283, S1996), TPR (S1185), and RANBP2 (T2613, S2805, S2280, S128, T1412), whereas negative co-regulation was observed with NUP188 (S1523). T106 showed positive co-regulation with NUP35 (T106, S105, T308), NUP98 (S1023), NUP133 (S50), NUP153 (S529, S522), AHCTF1 (S1898, S2222), RANBP2 (S128, S21, T2613, S2280), NDC1 (T414, S406), and NUP107 (S11), while negative co-regulation was observed with NUP98 (S1028) and NUP188 (S1523). Consistent with the known function of motif regions in nuclear localization, this co-regulation pattern suggests that phosphorylation at these sites may contribute to nucleocytoplasmic transport and subcellular localization of NCL.

In addition to nucleoporins, several phosphosites of the proliferation marker kinase Ki-67 (MKI67) and nucleophosmin 1 (NPM1) were repeatedly identified as co-regulated PsOPs of TPxKK motif-region-predominant phosphosites (T121, T76, T99, and T106), suggesting that these PsOPs may have functional significance. MKI67 is a key organizer of the perichromosomal layer and is required for the recruitment of nucleolar proteins, including NCL and NPM1, to the mitotic chromosome [27]. Previous studies have shown that at the chromosome periphery, distinct subcomplexes including NCL and NPM1 play a role in mitotic spindle regulation and kinetochore–microtubule attachment, thereby facilitating proper chromosome segregation during mitosis [28]. Collectively, these observations suggest that phosphorylation of predominant phosphosites within this region may have a potential role in mitotic progression, chromosome segregation, and nucleolar reorganization during cell division.

Predominant phosphosites within the RRM domain are primarily co-regulated with RNA-binding and RRM domain PsOPs, suggesting a functional association linked to RNA binding and RNA processing activities. S580 was positively co-regulated with several RRM domain-containing PsOPs, including NOL8 (S304), HNRNPA2B1 (S225, S189, S231), SNRNP70 (S410), NELFE (S249), HNRNPM (S365, S432, S481), MYEF2 (S17), SNRPA (S115, S197), HTATSF1 (S607, S702, S445, S579, S453, S597, S600), HNRNPA3 (S370), DNAJC17 (S112), SAFB2 (S287, S109, S207, S613), HNRNPL (S291, S589, S52), and HNRNPC (S299), whereas S619 was positively co-regulated with HNRNPD (T193, S241), HNRNPA0 (S188), EIF3B (S154), HNRNPA2B1 (S225, S341), SAFB2 (S195, S233), NELFE (S51), HNRNPM (S365, S701), HNRNPL (S101, S589), HNRNPA1 (S368), and HTATSF1 (S2). Many of these proteins are associated with mRNA processing, splicing, transport, and transcriptional regulation [29,30,31,32]. These findings indicate an association between RRM domain phosphosites and co-regulated PsOPs involved in RNA metabolism.

### 2.6. Identification of Known and Potential Upstream Kinases of Predominant NCL Phosphosites

Phosphorylation is a key regulatory mechanism that controls the activation of many proteins by protein kinases [33]. NCL is a highly phosphorylated protein, particularly at its N-terminus [5]. It serves as a substrate of multiple kinases, and its phosphorylation has been implicated in the regulation of diverse functions [9]. Specifically, AKT serine/threonine kinase 1 (AKT1) [34], protein kinase AMP-activated alpha catalytic subunit alpha 1 (AMPKA1) [35], CDK1 [36], cyclin-dependent kinase 2 (CDK2) [37], and cell division cycle 7 (CDC7) [38] have been reported as the regulatory kinases of NCL.

**Figure 4:**
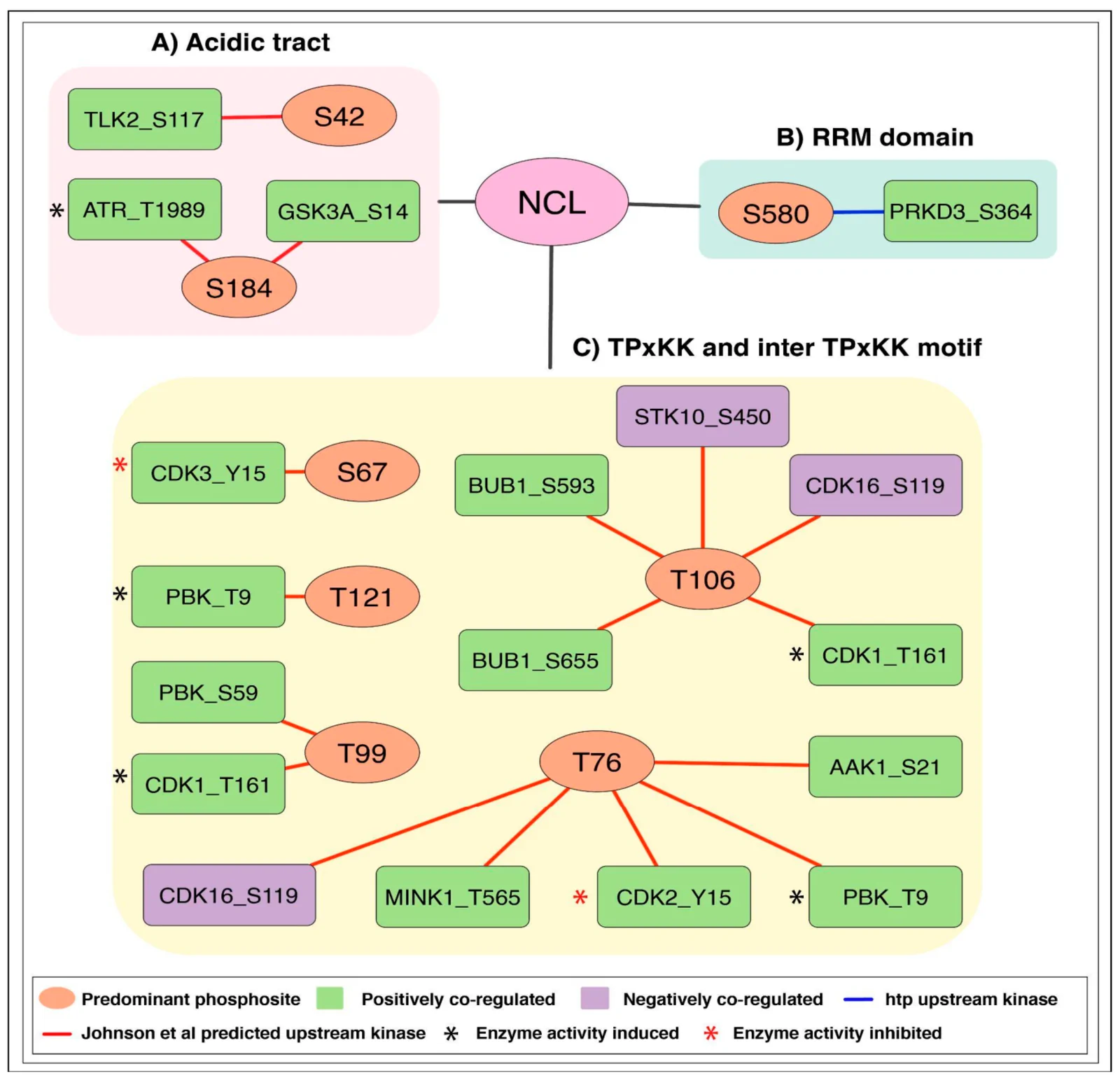
Network representation of potential upstream kinase phosphosites co-regulated with predominant phosphosites of NCL. (**A**) Upstream kinases co-regulated with the predominant phosphosites located within the acidic tract, (**B**) upstream kinases co-regulated with the predominant phosphosites within the RRM domain, and (**C**) upstream kinases co-regulated with the predominant phosphosites within and between the TPxKK motif.

In our analysis, several kinase phosphosites were found to be co-regulated with predominant phosphosites of NCL. To identify the potential upstream kinases co-regulated with these phosphosites, kinase–substrate motif specificity analysis based on datasets generated by Johnson et al. (2023) [39] was employed. Integration of co-regulation analysis with these approaches identified a few co-regulated kinases as candidate upstream kinases of predominant NCL phosphosites. The number of kinase phosphosites and upstream kinases co-regulated with predominant phosphosites of NCL are summarized in Table 2. The extent to which the predominant phosphosites exhibited co-regulation varied with individual sites. Though sites such as S28, S34, and S153 exhibited limited co-regulation with kinase phosphosites and no predicted upstream kinases, S41 and S145 exhibited no co-regulation with any kinase phosphosites or predicted upstream kinases under the defined selection criteria. Conversely, several other predominant phosphosites exhibited extensive co-regulation. Upstream kinases co-regulated with predominant phosphosites of NCL are illustrated in Figure 4 and Supplementary Table S4A. The positively and negatively co-regulated kinases of predominant NCL phosphosites are provided in Supplementary Table S4B.

Among the predominant phosphosites located in the acidic tract, S184 exhibited positive co-regulation with ATR checkpoint kinase (ATR) (T1989) and glycogen synthase kinase 3 alpha (GSK3A) (S14). ATR and GSK3A were identified as potential upstream kinases predicted by Johnson et al. (2023) [39]. ATR autophosphorylation at T1989 is a marker of ATR activation [40], and Kawamura et al. have shown that this autophosphorylation decreased in nucleolin-depleted cells [3], suggesting a potential functional association between NCL (S184) and ATR (T1989). S42 showed positive co-regulation with TLK2 (S117), which was identified as a potential upstream kinase by Johnson et al. (2023) [39]. The co-regulation of S42 with TLK2 (S117) is particularly significant, as it is known to play critical roles in DNA replication and repair, transcription, cell-cycle checkpoint recovery, chromatin maintenance, and genomic stability [41]. Moreover, TLK2 phosphorylates ASF1, facilitating its binding to histones and interaction with chaperone proteins, ensuring a proper histone supply during DNA replication [42]. Given that S42 is located within the histone-binding acidic tract region of NCL, the observed co-regulation pattern suggests a potential functional association between TLK2 (S117) and NCL (S42). In comparison to the predominant phosphosites clustered in the acidic region, the phosphosites in the TPxKK motif region exhibited extensive co-regulation with upstream kinases. Members of the CDK family, particularly CDK1, CDK2, and CDK16, were positively or negatively co-regulated with motif-region-predominant phosphosites. Particularly, CDK2 (Y15) and CDK16 (S119) co-regulated with T76; CDK1 (T161) co-regulated with T99; and CDK1 (T161) and CDK16 (S119) co-regulated with T106. It should be noted that the co-regulation of the predominant NCL sites with the kinase phosphosites does not establish a direct kinase–substrate association; moreover, phosphosites in kinases such as CDKs (inhibitory/activating phosphosites) were found to be co-regulated. A study by Liu et al. (2026) recently identified CDK2 as an upstream kinase of T76 and showed that TG-02 inhibited CDK2-mediated phosphorylation of T76 [43]. CDK2 (Y15) was consistently co-regulated with T76. However, phosphorylation at Y15 is well established to inhibit CDK2 activity. In addition, NCL is reported to be phosphorylated at its N-terminus by CDK1 during mitosis, and this differential phosphorylation may regulate NCL function during cell-cycle progression [4]. Furthermore, threonine phosphorylation of NCL by CDK1 has been associated with mitotic reorganization of nucleolar chromatin and facilitates its cytoplasmic translocation [44,45]. In addition, several additional predicted upstream kinases were identified in this region, including PDZ-binding kinase (PBK) (T9, S79) and BUB1 mitotic checkpoint serine/threonine kinase (BUB1) (S655, S593). These enzymes serve as key regulators of cell-cycle progression, mitosis and chromosome segregation [46,47]. Together with the co-regulation of motif-region-predominant phosphosites with nucleoporins, MKI67, NPM1, and other PsOPs involved in chromosome segregation, these findings indicate that the phosphorylation of predominant phosphosites within this region may coordinate NCL localization with cell-cycle-dependent processes.

Among the inter-motif phosphosites, S67 was positively co-regulated with cyclin-dependent kinase 3 (CDK3), which was phosphorylated at Y15, a phosphosite currently associated with the inhibition of its activity. CDK3 was identified as a predicted upstream kinase of NCL_S67 by Johnson et al. (2023) [39]. CDK3 belongs to the CDK family and acts as a regulator of the cell cycle by facilitating the G0-G1 and G1-S phase transitions [48], suggesting a potential role of these phosphosites in cell-cycle signaling pathways.

Among the RRM domain phosphosites, S580 was positively co-regulated with protein kinase D3 (PRKD3) (S364), a high-throughput upstream kinase. Members of the PKC/PKD kinase families are implicated in signal transduction, cell survival, proliferation, cell death and cancer-associated signaling [49,50]. In contrast, the inter-RRM phosphosite S563 showed limited kinase co-regulation with only one positively co-regulated kinase, and no potential upstream kinases. Taken together, these findings suggest that NCL phosphorylation is integrated with multiple signaling pathways governing chromatin organization, cell-cycle regulation, nucleolar reorganization, RNA processing, and tumor-associated signaling, highlighting the potential role of region-specific phosphorylation in multiple cellular processes.

### 2.7. Identification of Binary Interactors Co-Regulated with Predominant NCL Phosphosites

To examine the PsOPs that directly interact with the predominant phosphosites of NCL, its binary interactors were compiled from databases, including BioGRID, InAct, and BIND. Altogether, 199 interactors exhibiting positive or negative co-regulation with predominant NCL phosphosites were identified. Among these, several common interactors were found to be co-regulated with predominant phosphosites across different regions of NCL. Specifically, the predominant phosphosites T121, T76, T99 and T106 were positively co-regulated with six common interactors: anillin (ANLN) (S449), NPM1 (S139), mediator of DNA damage checkpoint 1 (MDC1) (T1800), VRK serine/threonine kinase 3 (VRK3) (S71), bromo domain-containing 7 (BRD7) (S289), and ribosomal L1 domain-containing 1 (RSL1D1) (T401). Specifically, NPM1, MDC1, VRK3, and BRD7 were involved in DNA repair [51,52,53]. NPM1 and NCL have previously been identified as the interacting partners of VRK3, and phosphorylation of these proteins might be regulated by VRK3 [52]. The co-regulation of NCL phosphosites with VRK3 (S71) and other DNA-repair-associated PsOPs supports a potential functional association between NCL phosphorylation of predominant phosphosites within this region and DNA damage response.

The inter-RRM phosphosite S563 exhibited limited co-regulation with only three PsOPs, namely MDC1 (S998), citron rho-interacting serine/threonine kinase (CIT) (S1993) and nuclear receptor subfamily 2 group C member 2 (NR2C2) (S19). These proteins are mainly associated with DNA damage responses, cytokinesis, and transcriptional regulation [54,55,56]. Notably, MDC1 is a key mediator of DNA damage signaling and has been reported to functionally associate with NCL, suggesting a potential role of S563 in genome stability-related processes [57]. In contrast, S580 and S619 exhibited extensive co-regulation with binary interactors and shared 14 common interactors such as serine and arginine repetitive matrix 1 (SRRM1) (S597), nibrin (NBN) (S518), ribosomal protein L3 (RPL3) (S13), Hect, UBA, and WWE domain-containing E3 ubiquitin protein ligase 1 (HUWE1) (S1368), Aly/REF export factor (ALYREF) (S239), bromo domain-containing 4 (BRD4) (S1117), chromodomain helicase DNA-binding protein 4 (CHD4) (T703), heterogeneous nuclear ribonucleoprotein U (HNRNPU) (S59), NR3C1 (S134), MCM2 (S108), SRSF3 (S5), CHD4 (T1653), HDGFL2 (S625), and ILF3 (S190). Among these, SRRM1 (S597), RPL3 (S13), ALYREF (S239), BRD4 (S1117), HNRNPU (S59), SRSF3 (S5), and ILF3 (S190) are involved in RNA processing, mRNA splicing, RNA transport, and translational regulation [58,59,60,61,62,63,64]. These observations suggest that phosphorylation of these phosphosites within the RRM domain may contribute to the coordinated regulation of the RNA metabolic pathway.

**Figure 5:**
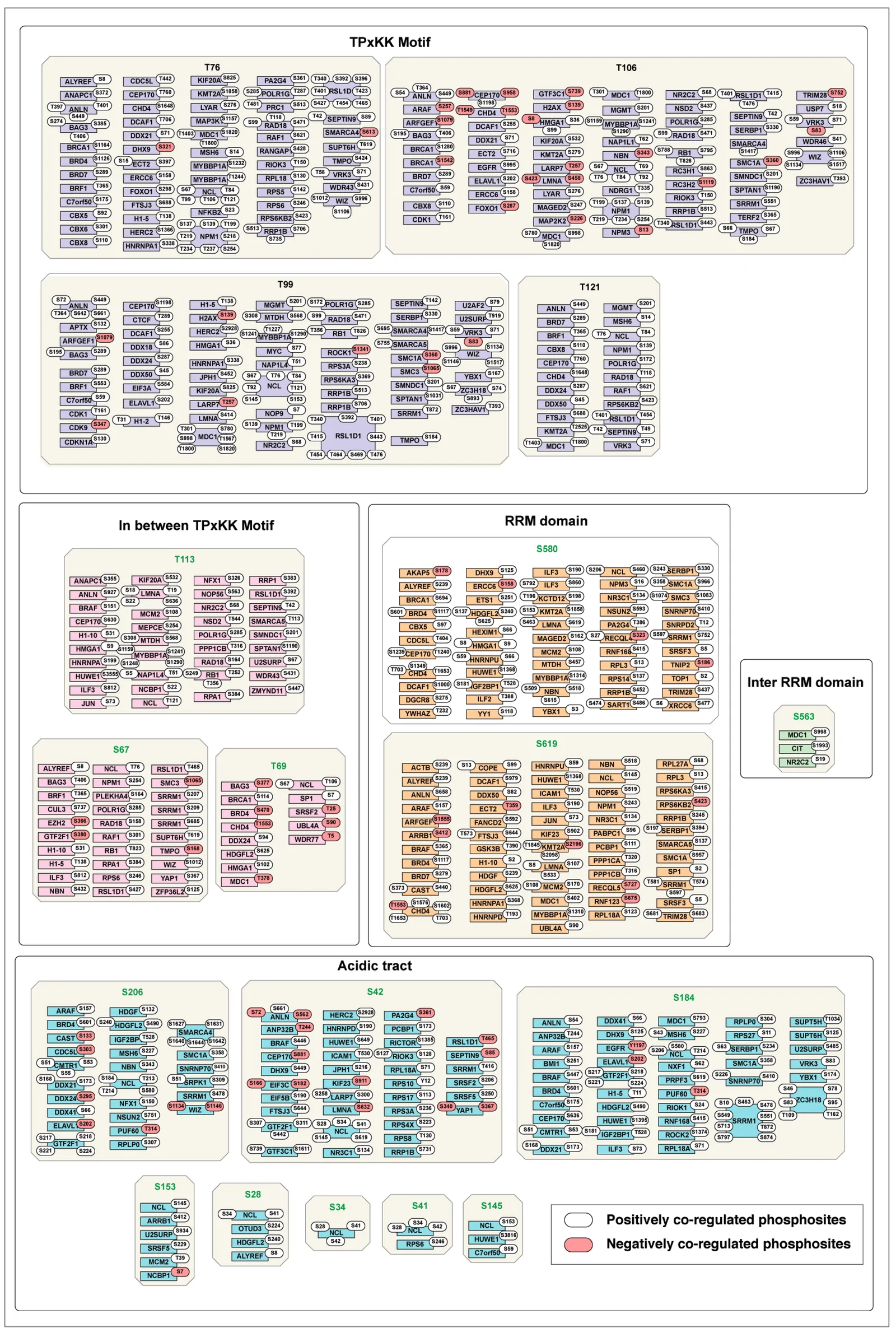
The diagram illustrates the positively and negatively co-regulated binary interactors associated with predominant phosphosites of NCL across different structural domains.

Overall, these observations highlight the plausible functional diversity of NCL phosphorylation and suggest that region-specific phosphorylation patterns may coordinate distinct cellular processes through a dynamic protein interaction network. The positively or negatively co-regulated binary interactors of predominant NCL phosphosites are provided in Figure 5 and Supplementary Table S5.

### 2.8. Differential Phosphorylation of the Predominant NCL Phosphosites Across Cancers

**Figure 6:**
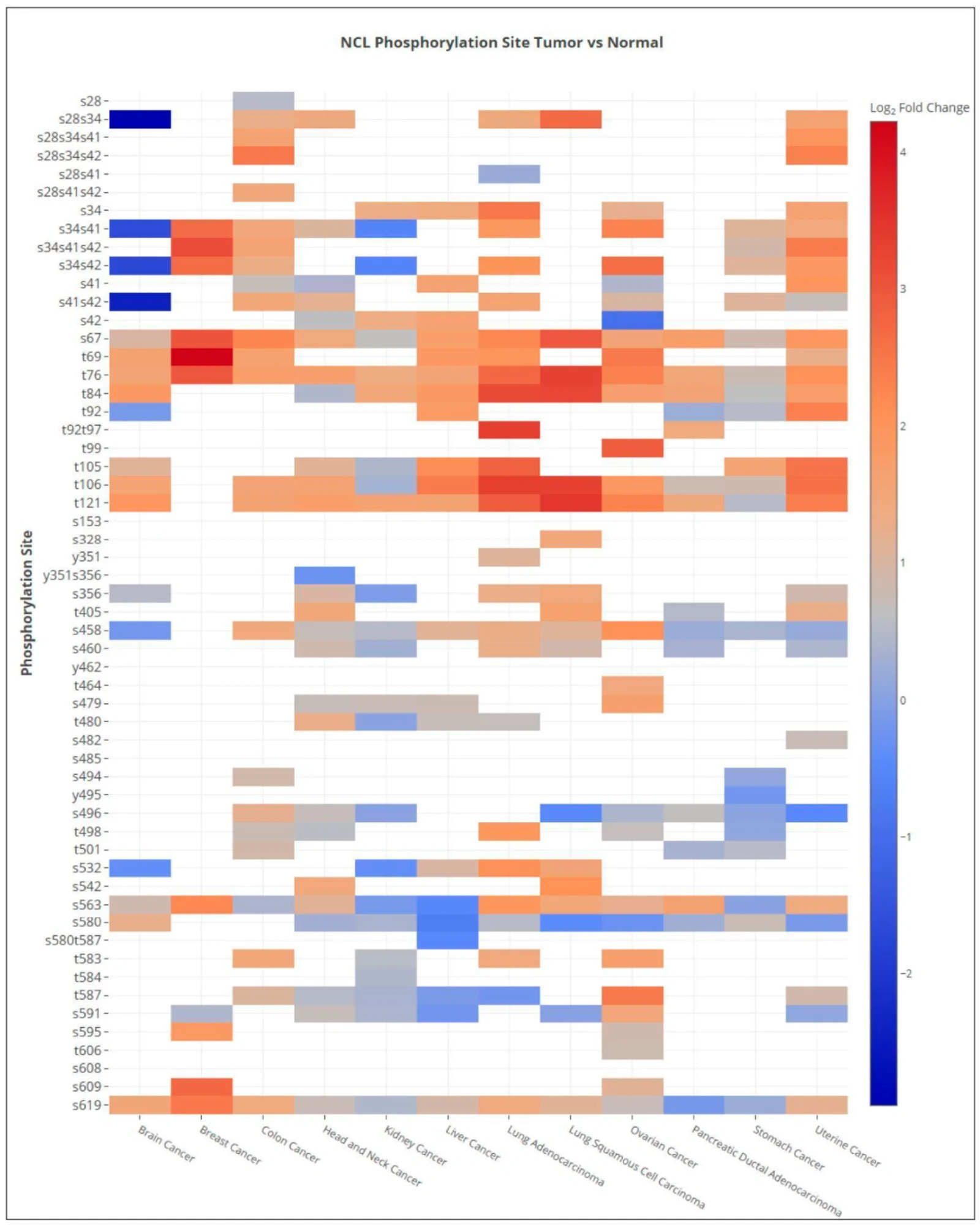
Differential phosphorylation profiles of NCL phosphosites across various cancer types from the cProsite database.

Aberrant expression and altered subcellular localization of NCL are frequently observed in cancers, where its altered distribution contributes to carcinogenesis, tumor cell proliferation, survival, metastasis and cancer progression [14,65]. Christian et al. demonstrated that NCL on the cell surface is overexpressed in several cancers and regulates tumor progression through interaction with specific ligands [66]. In addition, PRKCE- or CK2-mediated phosphorylation of NCL has been reported to enhance its interaction with Hsc70 and promote cell-surface localization, thereby contributing to angiogenesis and tumor progression [67]. The identification of PRKCE as a potential upstream kinase for the predominant phosphosites S184 and S619 located within the acidic tract and RRM domain, respectively, suggests a possible role of phosphorylation at these sites in cancer-associated regulatory pathways involving NCL localization, protein interactions, and pro-tumorigenic signaling. To delineate the possible role of predominant NCL phosphosites in cancer, we analyzed their phosphorylation pattern across tumor and normal tissue using the cProsite database. A heatmap illustrating site-specific phosphorylation profiles of NCL phosphosites is shown in Figure 6.

The cProsite database-based analysis of predominant NCL sites revealed a distinct cancer-specific phosphorylation pattern. Several phosphosites exhibited elevated phosphorylation across multiple cancers. Among these, inter-motif phosphosites S67 and T69, TPxKK motif phosphosite T76, RRM domain phosphosite S619, and acidic tract phosphosites S34, S41, and S42 showed increased phosphorylation in breast cancer. Previous studies have reported that NCL is overexpressed in breast cancers, particularly in aggressive forms such as triple-negative breast cancer (TNBC), at both mRNA and protein levels [68]. These findings indicate that the enhanced phosphorylation of these predominant phosphosites may be relevant to the modulation of the tumor-related pathways in breast cancer.

In contrast, acidic tract phosphosites S28, S34, S41, and S42 showed reduced phosphorylation in brain cancer, suggesting tumor-specific differences in NCL phosphorylation patterns. Increased phosphorylation of TPxKK motif-associated phosphosites (T121, T76, T99, T106) was also observed in cancers such as lung adenocarcinoma, lung squamous cell carcinoma, ovarian cancer and uterine cancer, indicating the possible role of this motif in tumor-specific functions of NCL.

The therapeutic potential of NCL to inhibit cancer progression has been investigated using several NCL-targeting agents, including the aptamer AS1411, F3 peptides, the multivalent pseudopeptide N6L and HB-19 [6]. Among these approaches, AS1411 is particularly relevant in the context of phosphorylation-dependent regulation of NCL. Iturriaga-Goyon et al. (2021) [69] have demonstrated that AS1411 treatment reduced the phosphorylation of NCL as detected using a phospho-T76-specific antibody in human umbilical vein endothelial cells (HUVECs). This decrease in NCL phosphorylation was associated with endothelial cell proliferation, migration, tube formation and angiogenesis [11,69]. These findings underscore NCL T76 as a potential phosphorylation site associated with NCL-mediated cellular functions.

Collectively, these observations indicate that targeting NCL and its potential phosphorylation-dependent functions could serve as a potential therapeutic strategy for modulating NCL-associated tumor and angiogenic processes.

## 3. Materials and Methods

### 3.1. Global Phosphoproteomic Screening and the Identification of NCL Phosphosites

We performed a PubMed search using the keywords “phosphoproteomics” OR “phosphoproteome” to identify the global phosphoproteomics datasets reporting NCL phosphosites. Review articles and plant-related studies were excluded, and our study considered only high-confidence phosphoproteomic datasets from human cell lines, which were manually curated from the published literature. The datasets were categorized on the basis of phosphopeptide enrichment strategies (STY/ST/Y phosphorylation sites). Stringent filtering criteria were used to identify high-confidence phosphosites, with sites achieving a localization probability of ≥75% and an A-score ≥ 13 classified as class-1 phosphosites. The datasets were further grouped into differential datasets, which compared phosphosite abundance between experimental conditions and their corresponding controls, and profile datasets, where phosphosite data from test and control samples were treated separately. Differential datasets were evaluated to identify regulatory changes based on statistical significance (*p* < 0.05) and fold change, and phosphosites with a fold change of ≥1.3 were considered to beupregulated, whereas those with a fold change of ≤0.76 were classified as downregulated. To maintain consistency among datasets, individual proteins were annotated according to the latest HUGO Gene Nomenclature Committee (HGNC) guidelines [70] and mapped to their corresponding UniProt accessions [71,72]. For NCL phosphosite annotation, residue numbering was based on the canonical human NCL sequence corresponding to the UniProt accession P19338. This canonical sequence was used consistently for phosphosite numbering across the integrated datasets.

### 3.2. Mapping of the Predominant NCL Phosphosites

Class-1 phosphosites assembled from the human cellular phosphoproteome were analyzed to determine the predominant phosphosites of NCL. For each phosphosite, the quantitative datasets in which it was differentially regulated were computed and ranked. Phosphosites meeting a frequency threshold of at least 20% of the top recurrently reported phosphosites in differential datasets and detected in a minimum of 15 experimental conditions were considered as the predominant phosphosites of NCL. Any phosphosites identified by phospho-specific antibodies or mutation-based approaches and not frequently reported as class-1 phosphosites in these datasets were excluded from this analysis.

### 3.3. Co-Occurrence Analysis of NCL Phosphosites

To analyze the mutual relationship among NCL phosphosites, a co-occurrence analysis was conducted based on the co-regulation pattern exhibited by the phosphosite pairs within NCL. From the differential datasets, the NCL phosphosites captured under the same experimental conditions were retrieved. For each phosphosite pair, the frequencies of UU, UD, DD and DU were calculated independently. The frequencies of the positive and negative co-regulation were calculated using the ratios ∑ (nUU + nDD)/∑ (nUD + nDU) and ∑ (nUD + nDU)/∑ (nUU + nDD), respectively. This approach enabled the evaluation of potential co-occurrence among phosphosites and their potential functional association within co-regulation networks [73].

### 3.4. Identifying Protein Phosphosites That Co-Regulated with the Predominant NCL Phosphosites

In order to determine the co-regulatory relationship between the predominant NCL phosphosites and phosphosites in other proteins (PsOPs), we sorted the differential regulation datasets associated with each phosphosite independently. Each predominant phosphosite was examined to determine its co-regulation with PsOPs. The quantitative differential datasets exhibiting upregulated phosphorylation of predominant NCL phosphosites were denoted as Ux, whereas those exhibiting downregulated phosphorylation were denoted as Dx. The PsOPs (denoted as o) displaying upregulation or downregulation in response to NCL (x) upregulation were categorized as UxUo and UxDo, respectively. Similarly, those showing corresponding upregulation or downregulation when NCL was downregulated were designated as DxUo and DxDo. The UxUo and DxDo categories were represented as positive co-regulation, while UxDo and DxUo were considered as negative regulation.

Using a contingency table, a one-sided Fisher’s exact test was carried out to identify the co-regulation between the predominant phosphosites of NCL and the associated PsOPs within the differential data. For each phosphosite pair, two one-sided Fisher’s exact tests were performed separately, one for positive co-regulation and another for negative co-regulation, to evaluate whether either pattern occurred more often than expected.

Fisher’s exact test (FET):

$$\sum p=\;\frac{\left(a+b\right)!\left(c+d\right)!\left(a+c\right)!\left(b+d\right)!}{n!}\;\sum_{i}\frac{1}{a_{i}!b_{i}!c_{i}!d_{i}!}$$

where a (n_0n0o) indicates the number of experimental conditions in which neither the NCL phosphosites nor the PsOPs were detected, b (n_Ux0 + n_Dx0 + n_0Uo + n_0Do) corresponds to the number of experimental conditions where only one of the two phosphosites was observed (either up or downregulated) while other remained undetected, c (n_UxDo + n_DxUo) denotes the number of experimental conditions exhibiting negative co-regulation between the two phosphosites, and d (n_UxUo + n_DxDo) indicates the number of experimental conditions where both sites displayed positive regulation. These values were used to construct a contingency table and Fisher’s exact test was applied to identify the PsOPs significantly co-regulated with predominant NCL phosphosites.

For further analysis, datasets were filtered using multiple criteria. Because each NCL phosphosite was evaluated against a larger number of phosphosites, the *p*-value obtained from the FET analysis was adjusted for multiple hypothesis testing using the Benjamini–Hochberg false discovery rate (FDR) procedure. The resulting q-value reflects FDR-adjusted significance levels for the individual hypothesis. The complete set of phosphosite-pair combinations was included in the analysis to ensure that multiple testing correction accounted for all hypotheses tested for each predominant NCL phosphosite. Phosphosite-pair associations with a q-value < 0.05 were considered statistically enriched. The magnitude of co-regulation was assessed using Odds Ratios with 95% confidence intervals to show the strength of the association between the phosphosites. In addition to statistical significance, PsOPs were required to satisfy the following criteria: (i) To identify consistently co-regulated phosphosite pairs, a minimum threshold of 10% of the total identification frequency of each predominant NCL phosphosite was applied separately to positive and negative co-regulation. The ratios were calculated as n(UxUoDxDo)/(UxDoDxUo) for positive co-regulation and n(UxDoDxUo)/(UxUoDxDo) for negative co-regulation. (ii) Support from at least three experimental conditions (experimental code count) showing positive or negative regulation. (iii) Evidence from at least three distinct studies (PubMed IDs) indicating positive or negative regulation. The PsOPs meeting these selection criteria were included for the downstream analysis to assess their potential roles in various biological processes, signaling mechanisms, protein–protein interactions and kinase–substrate associations [74].

### 3.5. Identification of Known and Predicted Upstream Kinases and Binary Interactors of NCL

The experimentally validated kinases of predominant NCL phosphosites and their specific phosphosites were obtained from PhosphositePlus [75], Phospho.ELM 9.0 [76], and RegPhos 2.0 [77]. The kinases predicted by synthetic peptide screening of kinome-wide substrate specificity using a 90th percentile cutoff were also included in this analysis [39]. Experimentally validated protein–protein interactors of NCL were obtained by integrating data from multiple databases, including HPRD [78], BIND [79], ConsensusPathDB [80], NetPath [81], and CORUM [82].

### 3.6. Data Visualization

To illustrate the data, lollipop plots were produced using M2Viz [83]. We used various Python (Version 3.14) packages such as Pandas (version 3.0.1) and Matplotlib (version 3.10.6) to visualize the distribution of phosphosites across the differential datasets. The interaction maps and networks were constructed using PathVisio 3 [84], Cytoscape (version 3.10.4) [85] and IBS 2.0 [86]. In addition, cProsite (https://cprosite.ccr.cancer.gov/), a web-based computational resource, was employed to identify the potential role of predominant NCL phosphosites in cancer [87].

## 4. Conclusions

This study provides a domain-specific phosphosite-centric view of NCL phosphorylation through the integration of large-scale phosphoproteomic datasets. By systematically identifying the predominant NCL phosphosites and integrating co-regulation analysis, upstream kinase prediction, and interaction network mapping, we characterized signaling networks associated with individual phosphosites of NCL and their potential functional significance. The identified predominant phosphosites exhibited distinct region-specific regulatory associations that were consistent with the established functions of NCL. Phosphosites within the acidic tract were primarily co-regulated with PsOPs involved in chromatin organization and histone-related processes; phosphosites within the TPxKK motif region were linked to nucleocytoplasmic transport, cell-cycle progression, chromosome segregation and nucleolar reorganization, whereas phosphosites within the RRM domain were associated with RNA-binding proteins and RNA metabolic processes.

Importantly, the clustering of predominant phosphosites within specific functional regions and their coordinated co-regulation patterns suggest that domain-specific phosphorylation may contribute to the regulation of distinct biological functions of NCL. The identification of known and potential upstream kinases, including members of the CDK, PKC, PKD, ATR and TLK2 families, further indicates that NCL phosphorylation is integrated into signaling pathways governing chromatin dynamics, DNA damage responses, cell-cycle regulation, RNA metabolism, nucleolar functions and oncogenic signaling. Moreover, the differential phosphorylation patterns of predominant phosphosites across multiple cancers indicate their possible significance in tumor-associated regulatory mechanisms. Together, these findings lay a foundational framework for future functional studies and highlight the need for experimental validation to establish the mechanistic significance of the multi-site phospho-signaling networks identified in this study.

### Limitations and Future Perspectives

The present study is solely based on data integration from the publicly available mass-spectrometry-based phosphoproteomic datasets; therefore, several limitations should be addressed. The integration of multiple studies may introduce heterogeneity in data because of the diversity in the sample sources, experimental conditions, mass-spectrometry platforms and data quality. Reanalysis of the data for normalization and batch-effect correction was not possible due to the vastness in the curated datasets, restricted access to the raw data and a unified reprocessing pipeline. To reduce potential biases, multiple selection criteria, including FET *p*-value, FDR, experimental code confidence and PMID confidence, were applied. Although these selection criteria were used to identify high-confidence co-regulated phosphosites, co-regulation does not by itself establish a direct functional relationship between NCL phosphosites and biological processes. Therefore, the identified co-regulatory relationship should be considered as a hypothesis-generating observation for further investigation and experimental validation by site-directed mutagenesis and in vitro kinase assays. Despite these limitations, this study provides a systematic view of NCL phosphorylation and its associated signaling networks and offers a foundation for future wet-lab studies to investigate potential phosphorylation-dependent functions of NCL and their relevance to NCL-targeted therapies.

## Figures and Tables

**Table 1:** Table containing the number of positively and negatively co-regulated high-confidence PsOPs corresponding to the respective predominant phosphosites located in different regions.

Regional Distribution of NCL	Predominant Phosphosites	Co-Regulation	No of Co-Regulated PsOPs
Acidic tract	S28	Positive	32
		Negative	5
	S41	Positive	14
		Negative	3
	S34	Positive	11
		Negative	4
	S145	Positive	25
		Negative	3
	S153	Positive	26
		Negative	4
	S206	Positive	391
		Negative	68
	S184	Positive	582
		Negative	47
	S42	Positive	337
		Negative	157
TPxKK motif	T121	Positive	259
		Negative	10
	T76	Positive	754
		Negative	50
	T99	Positive	668
		Negative	70
	T106	Positive	598
		Negative	210
Inter-TPxKK motif	S67	Positive	352
		Negative	26
	T69	Positive	58
		Negative	79
	T113	Positive	211
		Negative	19
RRM domain	S580	Positive	762
		Negative	64
	S619	Positive	564
		Negative	137
Between the RRM domain	S563	Positive	57
		Negative	2

**Table 2:** Table summarizing the number of kinase phosphosites and upstream kinases co-regulated with predominant NCL phosphosites.

Regional Distribution of NCL	Predominant Phosphosites	Co-Regulation	Number of Co-Regulated Kinase Phosphosites	Number of Co-Regulated Upstream Kinases
Acidic tract	S28	Positive	3	0
	S28	Negative	0	0
	S41	Positive	0	0
	S41	Negative	0	0
	S34	Positive	1	0
	S34	Negative	0	0
	S145	Positive	0	0
	S145	Negative	0	0
	S153	Positive	2	0
	S153	Negative	0	0
	S206	Positive	25	0
	S206	Negative	6	0
	S184	Positive	39	2
	S184	Negative	3	0
	S42	Positive	27	1
	S42	Negative	6	0
TPxKK motif	T121	Positive	12	1
		Negative	1	0
	T76	Positive	43	4
		Negative	4	1
	T99	Positive	17	2
		Negative	8	0
	T106	Positive	22	3
		Negative	21	2
Inter-TPxKK motif	S67	Positive	20	1
		Negative	5	0
	T69	Positive	1	0
		Negative	5	0
	T113	Positive	8	0
		Negative	1	0
RRM domain	S580	Positive	37	1
		Negative	4	0
	S619	Positive	35	0
		Negative	9	0
Between the RRM domain	S563	Positive	1	0
		Negative	0	0

## Data Availability

The original contributions presented in the study are included in the article/Supplementary Material; further inquiries can be directed to the corresponding author.

## Supplementary Materials

The following supporting information can be downloaded at: https://www.mdpi.com/article/10.3390/ijms27209132/s1.
