# Supplementary material for: Co-Regulation Analysis of Domain-Specific Phosphosites Reveals Insights into the Phosphoregulation-Directed Functional Diversity of Nucleolin
Source: Int J Mol Sci. 2026 Oct 15;27(20):9132. doi: 10.3390/ijms27209132 (PMC13705481; doi:10.3390/ijms27209132)
Supplement: Supplementary file 1 [file ijms-27-09132-s001.zip › Supplementary Figure S1.pdf]

**Title: Co-Regulation Analysis of Domain-Specific Phosphosites Reveals Insights into the Phosphoregulation-Directed Functional Diversity of Nucleolin**

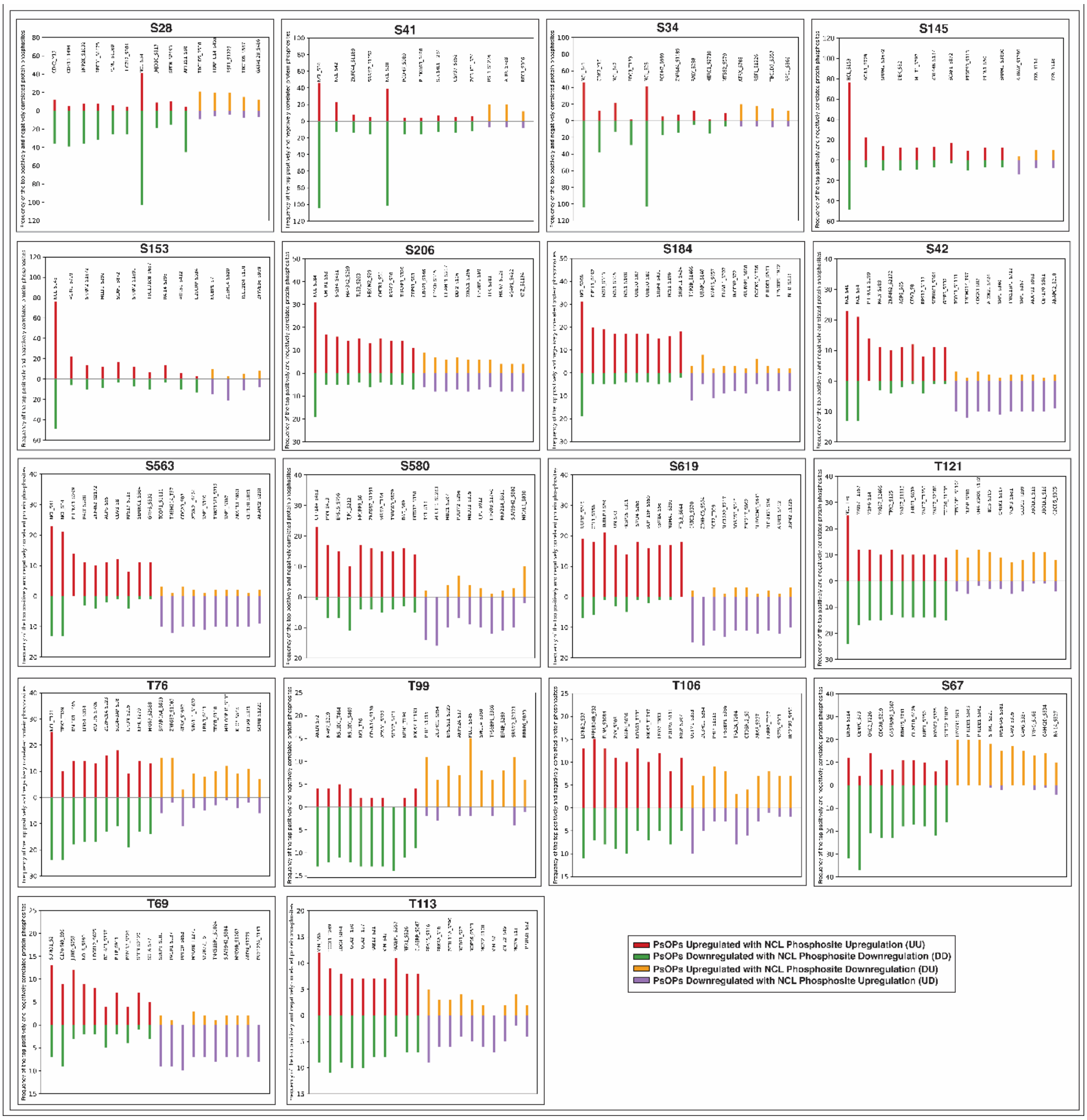


**Supplementary Figure S1:** Bar plot representing the top 10 high-confidence PsOPs that are positively or negatively co-regulated with predominant phosphosites of NCL.
